# 1012. Neutrophil Function Enhanced by Recombinant Interferon-Gamma in Newborns

**DOI:** 10.1093/ofid/ofab466.1206

**Published:** 2021-12-04

**Authors:** Ricardo Castillo-Galvan, Nicole Soper, Monique Bennett, Isaac Thomsen

**Affiliations:** 1 Karius, Inc., Franklin, Tennessee; 2 Vanderbilt University Medical Center, Nashville, Tennessee

## Abstract

**Background:**

Functional differences exist between neonatal and adult neutrophils. The incidence of infection is higher in preterm infants, and the severity of the immune impairment on the neonatal neutrophils is inversely related to gestational age. In order to recognize and combat life-threatening infections, neonates rely predominantly on the innate immune system.Neutrophils are an essential component of innate immunity, and they are the first responders against bacterial and fungal infections. Sepsis continues to be a prominent cause of neonatal mortality, especially among preterm infants. Recombinant interferon-gamma (IFNγ) effects on the immune system have included the upregulation of TLRs expression and stimulation of phagocytosis. They have been shown to reduce severe infections in children with chronic granulomatous disease.

**Methods:**

After the protocol was IRB approved, we enrolled term infants in their first 48 hours of life (Table 1). We then obtained free flow whole-blood samples through venipuncture from the cephalic vein. Samples were incubated with and without IFNγ for 24 hours. Isolation of unperturbed neutrophils using immunomagnetics was performed for a final concentration of 1x106/mL. We then assessed the neutrophil-bacterial interaction using fluorescent GFP-Staphylococcus aureus, and quantified neutrophil killing function on a novel assay involving fibrin matrix as a more physiologic and three-dimensional (3D) environment than standard in vitro or culture-based assays. We evaluated normalized progressive ratios, 20μL/80μL, 30μL/70μL, 40μL/60μL of Neutrophil/GFP-S aureus respectively.Table 1

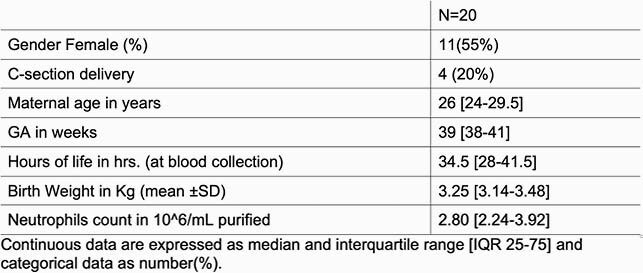

**Results:**

On the 20 samples, we observed significant differences demonstrating a considerably enhanced phagocytosis on those samples with the addition of IFNγ(p< 0.0001, Table 2 and Figures 1-3).

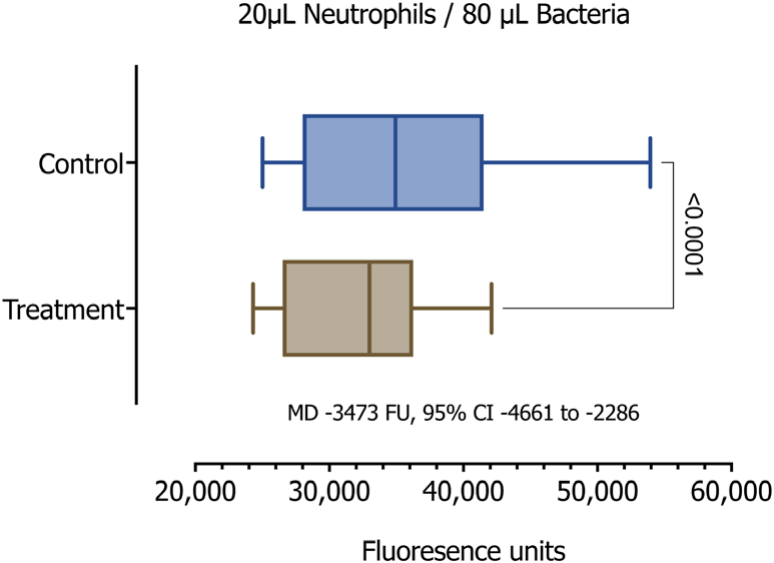

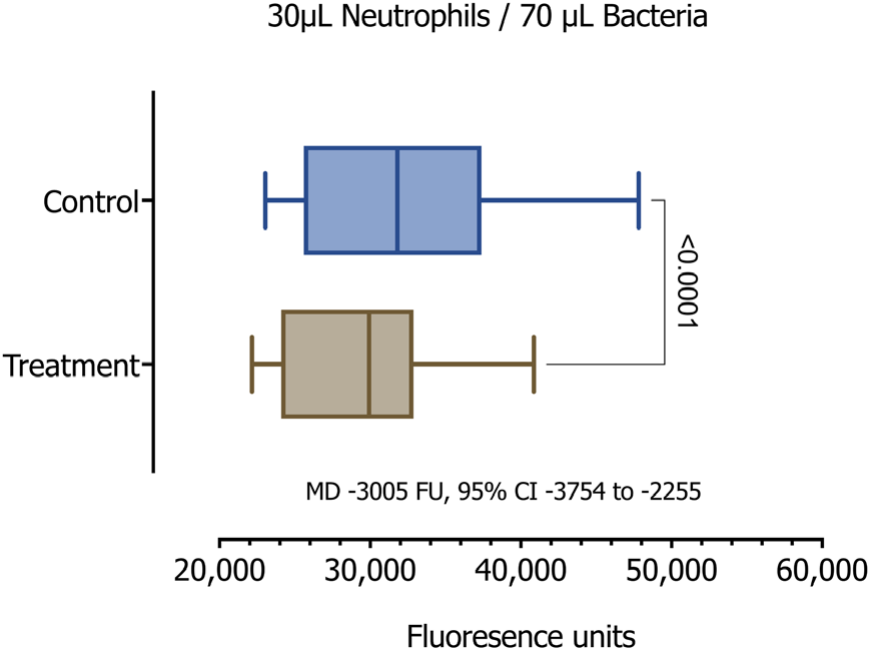

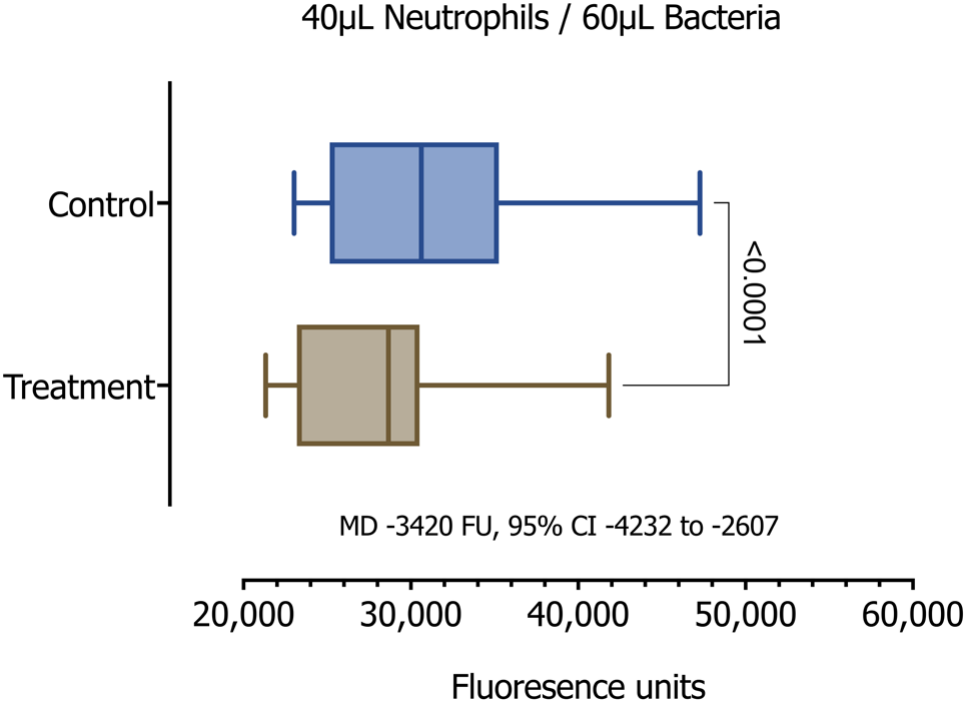

**Conclusion:**

The phagocytic ability of neonatal neutrophils was greatly enhanced by the addition of IFNγ in term infant blood. Ongoing work will determine whether this remains true for preterm-infant neutrophils and will further delineate mechanisms of these differences. We recognized an opportunity for interferon-based immunomodulation in certain situations on this population at high risk for invasive bacterial infections.

**Disclosures:**

**Ricardo Castillo-Galvan, MD MPH**, **Karius Inc.** (Consultant) **Isaac Thomsen, MD, MSCI**, **Horizon Therapeutics** (Consultant)

